# CXC-chemokine regulation and neutrophil trafficking in hepatic ischemia-reperfusion injury in P-selectin/ICAM-1 deficient mice

**DOI:** 10.1186/1476-9255-4-11

**Published:** 2007-05-24

**Authors:** Keith M Monson, Shadi Dowlatshahi, Elahé T Crockett

**Affiliations:** 1 Department of Physiology, College of Human Medicine, Michigan State University, East Lansing, Michigan, USA

## Abstract

**Background:**

Neutrophil adhesion and migration are critical in hepatic ischemia and reperfusion injury (I/R). P-selectin and the intercellular adhesion molecule (ICAM)-1 can mediate neutrophil-endothelial cell interactions, neutrophil migration, and the interactions of neutrophils with hepatocytes in the liver. Despite very strong preclinical data, recent clinical trials failed to show a protective effect of anti-adhesion therapy in reperfusion injury, indicating that the length of injury might be a critical factor in neutrophil infiltration. Therefore, the aim of this study was to assess the role of P-selectin and ICAM-1 in neutrophil infiltration and liver injury during early and late phases of liver I/R.

**Methods:**

Adult male wild-type and P-selectin/ICAM-1-deficient (P/I null) mice underwent 90 minutes of partial liver ischemia followed by various periods of reperfusion (6, 15 h, and a survival study). Liver injury was assessed by plasma level of alanine aminotransferase (ALT) and histopathology. The plasma cytokines, TNF-α, IL-6, MIP-2 and KC, were measured by ELISA.

**Results:**

Reperfusion caused significant hepatocellular injury in both wild-type and P/I null mice as was determined by plasma ALT levels and liver histopathology. The injury was associated with a marked neutrophil infiltration into the ischemic livers of both wild-type and P/I null mice. Although the levels of ALT and neutrophil infiltration were slightly lower in the P/I null mice compared with the wild-type mice the differences were not statistically significant. The plasma cytokine data of TNF-α and IL-6 followed a similar pattern to ALT data, and no significant difference was found between the wild-type and P/I null groups. In contrast, a significant difference in KC and MIP-2 chemokine levels was observed between the wild-type and P/I null mice. Additionally, the survival study showed a trend towards increased survival in the P/I null group.

**Conclusion:**

While ICAM-1 and P-selectin does not appear to be critical for neutrophil infiltration and I/R injury in the liver, they may regulate CXC-chemokine production. Blockage of these adhesion molecules may improve survival and remote organ injury that often accompanies liver I/R injury, through chemokine regulation.

## Background

Hepatic I/R injury can result from surgical resection or transplantation of the liver, from portal triad cross-clamping for control of hemorrhage in hepatic trauma, or after hemodynamic shock. In these situations, after a period of ischemia, the liver can be significantly injured upon its reperfusion [1]. If the injury is severe enough, this can lead to liver failure, systemic inflammatory response syndrome, acute respiratory distress syndrome, and multiple organ dysfunction syndrome, which are all associated with high rates of morbidity and mortality.

Hepatic I/R injury occurs in a biphasic pattern: The acute injury phase is characterized by hepatic injury occurring within 1–6 h after reperfusion, associated with Kupffer cell activation, and generation of reactive oxygen species (ROS) and the pro-inflammatory cytokines [2,3]. This is followed by the subsequent subacute-phase response that is characterized by a massive neutrophil infiltration, peaking 9–24 h following reperfusion. Neutrophil adhesion and migration is dependent on selectins, β 2 integrins (i.e., CD18: Mac-1, LFA-1) and members of the immunoglobulin gene superfamily adhesion molecules such as ICAM-1 [4-6]. The adherence of neutrophils to hepatocytes can be mediated by Mac-1/ICAM-1, Mac-1/unknown ligand(s) and lymphocyte function-associated antigen (LFA-1)/ICAM-1 [4-6].

Studies of endotoxin-induced liver injury have suggested an adherence-dependent neutrophil induced hepatocyte injury [7], while others indicated an adherence-independent cytotoxicity of the hepatocytes [8]. P-selectin and ICAM-1 involvement in neutrophil infiltration and I/R injury has been documented in several studies [9-13]. Likewise, other studies have reported a lack of significant role of these adhesion molecules in the liver I/R injury [7,13-15]. Additionally, recent clinical trials of anti-adhesion therapy in an attempt to reduce injury associated with traumatic shock and reperfusion injury failed to show a significant benefit, despite very strong preclinical data [16]. In an effort to understand the disparity between the preclinical and clinical trial studies, it was noted that the lengths of injury in the clinical setting were longer than those of the preclinical studies. It appears that the underlying mechanism of neutrophil infiltration with a short period of insult is different from those of injury associated with a longer period of insult. Therefore, we examined the role P-Selectin and ICAM-1 in liver reperfusion injury, at various lengths of reperfusion time. In the current study, we sought to test whether or not hepatic I/R injury would be attenuated in P/I null mice after longer periods of reperfusion, at time points most consistent with the neutrophil-mediated phase of liver injury.

## Methods

All chemicals were purchased from Sigma Chemical (St. Louis, MO), unless otherwise noted.

### Animals

Adult male mice (i.e., 8–10 wk) were used in this study. All animals received humane care in compliance with the *Guide for the Care and Use of Laboratory Animals *(National Institutes of Health Publication No. 85-23, revised 1985). Experimental protocols were approved by the Michigan State University Animal Use and Care Committee.

Gene-targeted double mutant mice deficient in P-selectin and ICAM-1 (P/I double mutant), C57BL/6-Icam1^tm1Bay^Selp^tm1Bay^, were used in this study. The breeding pairs of double-knockout mice were purchased from Jackson Laboratory (Bar Harbor, ME) and bred under the guidance of University Laboratory Animal Resources at Michigan State University. The wild-type mice were male C57BL/6. Before and after surgery, all the animals had unlimited access to food and water.

A murine model of lobar hepatic ischemia, as previously described by our laboratory, was used [13]. The experimental procedures were performed under aseptic conditions. Adult male mice (8–10 wk) weighing between 23–30 g were anesthetized with inhaled methoxyflurane (Baxter Caribe, Inc., Guayama, PR) followed by an intraperitoneal injection (35 mg/kg body wild-type) of sodium pentobarbital (Abbott Laboratories, North Chicago, IL). A midline laparotomy was performed. The ligamentous attachments of the left lateral and median lobes were carefully divided. The left lateral and median lobes were freed. The portal circulation to both of these lobes was carefully dissected and the portal vein and hepatic artery supplying the median and left lateral lobes were then interrupted with an atraumatic vascular clamp (Accurate Surgical and Scientific Instruments Corporation, Westbury, NY). The left lateral lobe was also rotated 180 degrees counter-clockwise on its vascular pedicle to eliminate any potential perfusion that might occur with an imperfect clamp occlusion. The caudate and right lateral lobes, as well as the papillary and quadrate processes, retained an intact portal and arterial inflow and venous outflow to prevent intestinal venous congestion. This procedure resulted in the induction of ischemia to approximately 65–70 percent of the liver. The mortality due to the surgical procedure was minimal (< 1–2%). After 90 minutes of partial hepatic ischemia the clamp was removed, the left lobe was rotated back 180 degrees clockwise, and reperfusion was initiated. The midline laparotomy was closed in a single layer fashion using 5-0 nylon suture. Sterile lactated Ringer's solution (0.8 ml) was administered subcutaneously to compensate for operative blood and fluid losses. Animals were divided into two groups; the test group underwent I/R and the sham group underwent the same anesthesia and midline laparotomy dissection of the portal vessels and liver, but without vascular occlusion. Mice were euthanized after 6 and 15 h of reperfusion and the blood and liver tissue were collected and processed, as described below. Additionally, a survival study was carried on in which the length of survival from the start of reperfusion was recorded up to three weeks at the time the mice were euthanized.

### Peripheral blood and tissue procurement

Blood samples were collected from the right ventricle via a left anterior thoracotomy in a sterile heparinized syringe containing 50 μl of heparin (100 USP Units/ml). The blood samples were centrifuged and plasma were collected and stored at -30°C until further use. Portions of the ischemic and non-ischemic liver lobes were fixed in buffered 10% formalin, embedded in paraffin, and used for hematoxylin and eosin (H&E) staining. Other portions of ischemic and non-ischemic liver lobes were snap frozen in liquid nitrogen and stored at -70°C, until use for immunohistochemistry staining, and MPO analysis.

### Demonstration of hepatocellular injury by determination of plasma alanine aminotransferase levels

The plasma ALT levels were determined spectraphotometrically, as previously described [13]. The ALT values are expressed in international units per liter (IU/L).

### Histopathological studies

H&E staining was performed on tissue sections prepared at 5-μm intervals. A pathologist, blinded to the experimental procedure of the mice, examined the histopathology of the hepatic tissue sections.

### Immunohistochemistry for ICAM-1 expression and neutrophil sequestration

ICAM-1 expression of the hepatic tissue was detected by an immunohistochemistry technique as previously published by our laboratory [13]. Briefly, cryosections (5-um thick) from ischemic and nonischemic hepatic lobes fixed in acetone were stained using an anti-mouse ICAM-1 antibody (3E2, IgG, Pharmingen, San Diego, CA) and a biotin-conjugated goat anti-hamster IgG secondary antibody (Pharmingen). ICAM-1 molecules were visualized using a Vectastain avidin-biotin complex reagent and 3,3'-diaminobenzidine chromogen kits (Vector Lab, Inc., Burlingame, CA). The tissue sections were examined using a Nikon light microscope interfaced with a Spot 24-Bit Digital Color Camera. Similarly, immunohistochemical staining for neutrophils was performed using a primary antibody (IgG_2a_) specific to the mouse neutrophil (Cedarlane International Distributor, Ontario, Canada).

### Plasma cytokine concentrations

Plasma TNF-α, IL-6, KC, and MIP-2 levels were determined in a 96-well Nunc-Immuno microplate (VWR Scientific, Chicago, IL), using a sandwich enzyme-linked immunosorbent assay (ELISA) technique, as previously described [17]. The capture antibody was a polyclonal anti-mouse TNF-α, IL-6, KC, or MIP-2 specific goat IgG (R&D Systems, Minneapolis, MN) and the detection antibody was a biotinylated polyclonal anti-mouse TNF-α, IL-6, KC or MIP-2 specific goat IgG, (R&D Systems). All plasma samples were tested in duplicate. The minimal detectable protein concentration was 20 pg/ml.

### Demonstration of neutrophil recruitment by myeloperoxidase (MPO) assay

Liver MPO content was measured according to the previously published method by our laboratory [17]. Briefly, the frozen liver tissues were homogenized using a Tissue Tearor, centrifuged and the pellets were resuspended in the buffer. The MPO activity was determined using a tetramethylbenzidine substrate kit (ImmunoPure, Pierce, Rockford, IL) and read at 450 nm using a human MPO as a standard. One unit of MPO activity was defined as the quantity of enzyme degrading 1 μmol peroxide/min at 25°C.

### Statistical analysis

All data are expressed as means ± SEM. Comparison between two groups was performed using an unpaired Student *t*-test. Comparisons between multiple groups and various time points were performed using a Kruskal-Wallis One-Way Analysis of Variance (*ANOVA*) followed by a Bonferroni test. Survival data was assessed using the Kaplan-Meier log rank test. Analysis was performed using the Number Cruncher Statistical System (Number Cruncher Statistical Systems, Kaysville, UT). *P *≤ 0.05 was considered significant.

## Results

### Verification of ICAM-1 deficiency in P/I null mice

**Figure 1:**
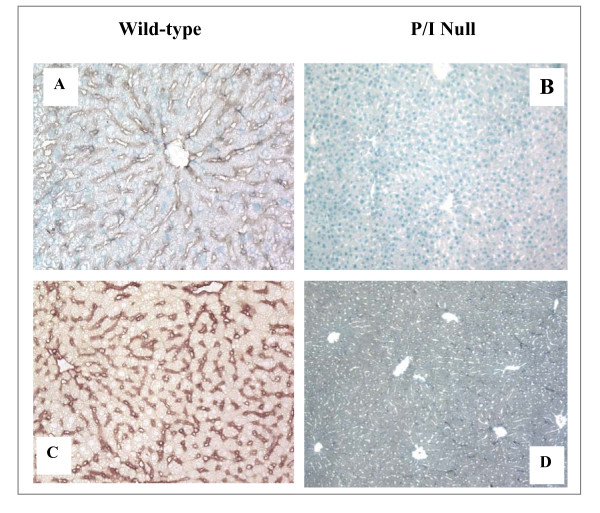
**Immunohistochemical Staining of Liver for ICAM-1 Expression**. Positive ICAM-1 staining (brown color) is demonstrated in wild-type mice along the vasculature and sinusoids, but is notably absent in the P/I null mice. (**A**) Control, wild-type mouse, (**B**) Control, P/I null mouse, (**C**) Wild-type mouse after 90 minutes of ischemia and 6 h of reperfusion, (**D**) P/I null mouse after 90 minutes of ischemia and 6 h of reperfusion.

The absence of ICAM-1, and P-selectin expression in the P/I null mice was confirmed in randomly selected litter mice by specific immunohistochemical staining and reverse transcriptase polymerase chain reaction (RT-PCR), as previously published by our laboratory [13]. The ICAM-1 expression was determined in all the animals used in this study. ICAM-1 was constitutively expressed in the wild-type control mice as indicated by brown staining along endothelial lining of the sinusoids, and hepatic vasculatures (Figure 1A). The ICAM-1 expression was markedly increased in wild-type mice following hepatic I/R (Figure 1C). In contrast, in P/I null liver tissue, ICAM-expression was absent (Figures 1B and 1D). The RT-PCR data are not shown.

### Demonstration of hepatocellular injury by changes in plasma ALT levels

**Figure 2:**
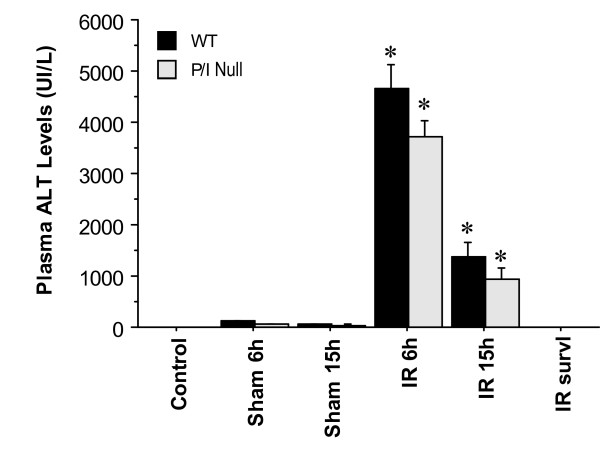
**Time-course of plasma ALT levels following hepatic I/R**. Mice were subjected to 90 minutes of ischemia followed by reperfusion with various lengths of time. "Control" indicates mice that underwent no surgical procedure. "Sham" indicates mice that underwent surgical procedure with no vascular occlusion followed by reperfusion, while "I/R" denote mice that underwent I/R surgical procedure. Values are expressed as mean ± SEM. (*) Sham-operated mice were statistically different from the I/R groups (i.e., *p *≤ 0.05). Sham and P/I group (n = 3–16 mice per each data time point).

Hepatic I/R caused significant hepatocellular damage as demonstrated by plasma ALT levels. The plasma ALT levels of both wild-type and P/I null mice after 90 minutes of ischemia followed by 6 and 15 h of reperfusion were significantly elevated when compared to their respective sham-operated mice (Figure 2). ALT levels were negligible in both groups by the survival time-point. Although there was no statistically significant difference in ALT levels between the wild-type and P/I null mice at either time point of reperfusion (i.e. 6 and 15 h), P/I null mice showed decreased ALT levels compared to the wild-type mice.

### Demonstration of hepatocellular injury by histopathology

**Figure 3:**
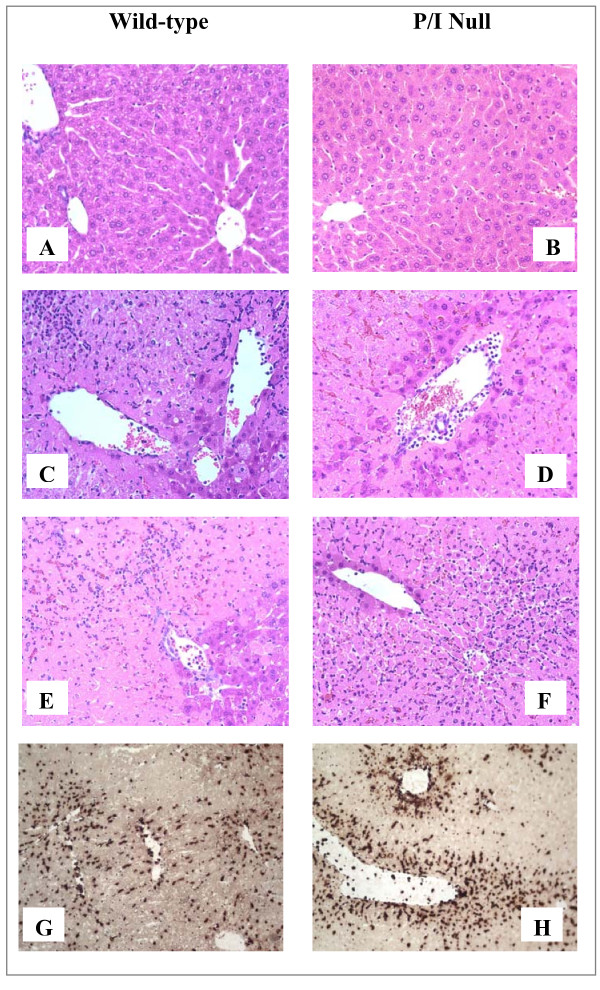
**Hepatic histopathology following I/R**. Wild-type and P/I null mice subjected to the sham operation or 90 minutes of liver ischemia followed by various reperfusion times. The ischemic liver sections were prepared and stained with H&E. Figures **A **and **B **represent the sham mice; there is essentially normal hepatic histology; and **C**, **D**, **E**, and **F **represent mice subjected to I/R. A pattern of reperfusion damage is evident by loss of hepatocytes in the pericentral and midzonal regions, with relative sparing of the periportal areas. Note the presence of neutrophils in the midzonal region around the central vein. Figures **G **and **H **show immunohistochemical staining of neutrophils using a specific anti-neutrophil antibody, i.e., subjected to 90 minutes of ischemia followed 6 h of reperfusion. Neutrophils are indicated by dark brown color stain. (**A**) Wild-type mouse, 6 hour sham; (**B**) P/I null mouse, 6 hour sham; (**C **and **E**) Wild-type mice subjected to 90 minutes of ischemia followed by 6 and 15 h of reperfusion respectively; (**D **and **F**) P/I null mouse subjected to 90 minutes of ischemia followed by 6 and 15 h of reperfusion, respectively.

**Figure 4:**
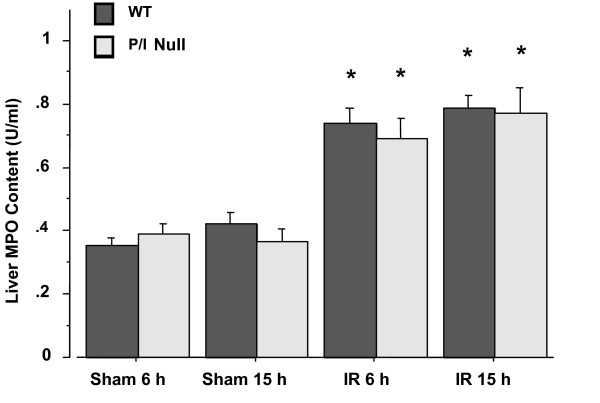
**Hepatic Myeloperoxidase Levels following I/R in Wild-type and P/I null mice**. Mice were subjected to the sham operation or to 90 minutes of liver ischemia with various reperfusion times (i.e., 6, and 15 hrs). The ischemic liver was collected and its MPO content was determined for the reperfusion time points indicated. Values are expressed as the mean ± SEM. (*) Sham-operated mice were statistically different from the I/R groups (i.e., *p *≤ 0.05). Comparison between Wild-type and P/I null at each time points indicated no significant differences.

The histopathologic injury of the liver tissue was evaluated based on sinusoidal congestion, cytoplasmic vacuolization, hepatocellular necrosis, and neutrophil infiltration. The liver sections from the sham-operated mice displayed minimal/no necrosis, similar to that of the non-operated control mice (Figure 3A and 3B). Additionally, there was no apparent evidence of hepatic injury due to ischemia alone (i.e., at zero hour of reperfusion; image not shown). However, reperfusion of the ischemic liver induced an extensive hepatocellular necrosis, sinusoidal congestion, and neutrophil infiltration after 6 and 15 h of reperfusion in both wild-type and P/I null mice (Figures 3C, 3D, 3E, and 3F). There was sparing of the periportal areas with progressively increased injury approaching the central vein. In general, it appeared that the wild-type I/R mice exhibited larger areas of coagulative necrosis when compared to the P/I null mice. The injury was associated with a marked number of neutrophils infiltrated into the midzonal region of ischemic liver after 6 and 15 h of reperfusion in both wild-type and P/I null mice, which was confirmed with in situ immunohistochemical staining of the neutrophils (Figure 3, last row). There were a minimal number of neutrophils present in the livers of sham-operated mice and in the non-ischemic lobes of mice subjected to I/R, indicating that ischemia was a pre-requisite for reperfusion injury to occur. Further, neutrophil infiltration was quantitated by measuring the liver MPO content (Figure 4). The liver MPO levels of both wild-type and P/I null mice after 90 minutes of ischemia followed by 6 and 15 h of reperfusion were significantly elevated compared to the sham-operated mice at the corresponding time points. Further, there was no statistically significant difference in MPO levels between the wild-type and P/I null mice, at either time point.

### Plasma TNF-α, IL-6, MIP-2, and KC levels

**Figure 5:**
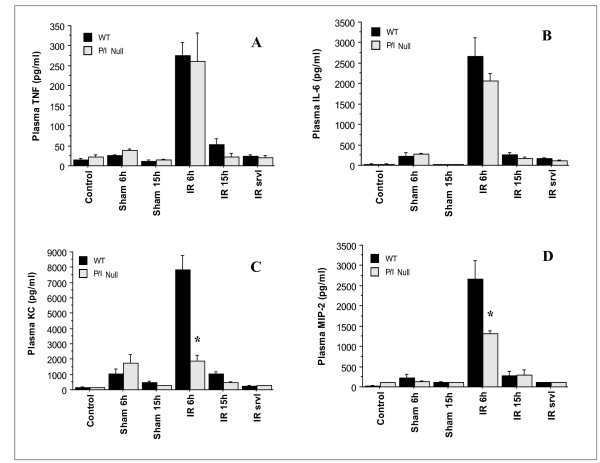
**Time-course of plasma TNF-α, IL-6, KC, and MIP-2 levels following various reperfusion times, after the onset of 90 minutes of ischemia**. "Control" indicates mice that underwent no surgical procedure. "Sham" indicates mice that underwent surgical procedure with no vascular occlusion followed by reperfusion, while "I/R" indicates mice that underwent surgical procedure with vascular occlusion for 90 minutes followed by reperfusion for various lengths of time. Values are expressed as mean ± SEM. *P < 0.05 wild-type group vs. P/I group (n = 3–16 mice per each time point/group). **A**: TNF-α data; **B**: IL-6 data; **C**: KC data; **D**: MIP-2 data.

In order to determine whether plasma cytokine/chemokine levels correlated with tissue injury, the plasma cytokine/chemokine levels were measured using an ELISA. As figure 5 displays, the plasma levels of all cytokines (i.e., TNF-α, IL-6, MIP-2, and KC) were significantly increased in response to I/R, in both the wild-type and P/I null groups, and reached their maximum at 6 h of reperfusion, which then declined to baseline by 15 h of reperfusion. The data related to the wild-type is consistent with our previous studies, where a similar pattern was observed in wild-type mice subjected to hepatic I/R [20].

As Figure 5A. shows, hepatic IR caused significant production of TNF-α, by 6 h of reperfusion in both wild-type and P/I mice, which declined by 15 h of reperfusion. However, the data indicates no significant difference in I/R-induced TNF-α production between the wild-type and P/I mice. This data is consistent with plasma ALT data, which showed a maximal increase in ALT levels at I/R 6 h, followed by a decrease in levels by I/R 15 h of reperfusion. A similar pattern was observed in plasma IL-6 levels (Figure 5B). In contrast, chemokine production showed a different pattern, in that the P/I null mice had significantly lower levels of plasma KC and MIP-2 at I/R 6 h than the wild-type mice (Figure 5C. and 5D).

### Survival study

**Figure 6:**
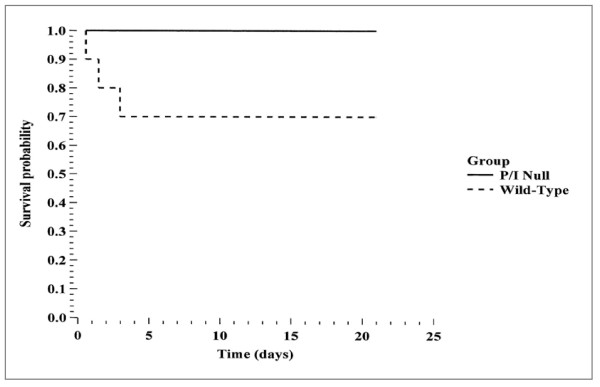
**Kaplan-Meier Survival Curve**. Mice were subjected to 90 minutes of liver ischemia followed by a 3-week period of reperfusion. The plot shows two curves. The solid line represents the survival curve for the P/I null mice (10/10 mice survived) and the broken line represents the survival curve for the wild-type mice (7/10 mice survived). Wild-type survival was not significantly different from P/I null survival (*p *= 0.067) although a trend is apparent.

Ten wild-type and ten P/I null mice were subjected to 90 minutes of partial hepatic ischemia and were observed post-operatively and their approximate times of death were recorded. At the end of three weeks, all surviving mice were euthanized. All the P/I null mice survived the full three weeks while only 7 out of 10 of the wild-type mice survived that length of time. The Kaplan-Meier log rank test showed no statistically significant difference between the two groups (p = 0.067), although a trend towards improved survival in the P/I null group was apparent (Figure 6).

## Discussion

Neutrophil infiltration plays an important role in reperfusion tissue injury, which is mediated by adhesion molecules such as selectins, β2-integrins, and ICAM-1. It has been suggested that inhibition of the adhesion molecules would prevent neutrophil infiltration, thus providing protection against organ injury caused by I/R. Currently though, there is a disparity between preclinical and clinical trial data, and it has been suggested that this disparity may be the result of the length of insult used in previous studies. Thus, the current study examined the role of P-selectin and ICAM-1, adhesion molecules involved in cytokine production, neutrophil infiltration, and hepatocellular injury, following hepatic I/R injury after short and longer periods of insult. Transgenic P/I null and wild-type mice were subjected to 90 minutes of warm liver ischemia followed by various periods of reperfusion. Hepatic I/R caused significant hepatocellular injury at 6 and 15 h of reperfusion in both wild-type and P/I null mice, which was associated with a marked increase in neutrophil infiltration to the ischemic liver. The difference between the two mouse groups was moderate and statistically insignificant. In contrast, there was a significant difference in CXC-chemokine production in that the P/I null mice had significantly lower levels CXC-chemokines than their wild-type mice counterparts. Additionally, P/I null mice showed a favorable trend to survival. These findings suggested that while P-selectin and ICAM-1 do not play a critical role for neutrophil infiltration and liver injury, it may regulate chemokine production and confer a survival advantage.

The data of the present study is consistent with previously reported studies that demonstrated no attenuation of neutrophil infiltration in hepatic sinusoids despite blocking a number of different adhesion molecules [14,18-20]. Studies have also shown that neutrophil infiltration was largely independent of the adhesion molecules, despite the presence of adhesion molecules on endothelial cells lining the hepatic sinusoids and vasculature [21,22]. In contrast, other studies have shown that neutrophil infiltration was dependent on the adhesion molecules and that hepatocellular injury was reduced by anti-adhesion antibody treatment [10,21]. These studies collectively indicate that the role of adhesion molecules is tissue and stimulus specific. As discussed below, there are a number of possible explanations as to why P-selectin and ICAM-1 deficiency did not appear to be critical for neutrophil infiltration and hepatocellular injury following liver I/R.

Although P-selectin is considered a critical adhesion molecule in initial tethering and rolling of neutrophils on endothelial cells, several studies suggest that P-selectin is unlikely to play an important role in hepatic injury through neutrophil sequestration or transendothelial migration. First, P-selectin is not expressed on the sinusoidal endothelium [22,23], where the predominant neutrophil extravasation takes place in the liver [7]. Second, within the liver venules, leukocytes can use other adhesion molecules such as α-4 integrin, independent of the selectins, and finally, within the liver sinusoids, no known selectin molecules or α-4 integrin molecules appear to play a dominant role in leukocyte recruitment [24]. Nevertheless, it should be noted that P-selectin might participate in I/R injury through its role in platelet aggregation and binding to the neutrophils [25]. Other factors such as swelling of the endothelial lining cells, vasoconstriction of the sinusoids, and, stiffening and decreased deformability of the neutrophils, may also contribute to the mechanical trapping of neutrophils in hepatic sinusoids. [26,27].

The study presented in this article suggests an ICAM-1 independent mediated neutrophil infiltration into the ischemic liver, though it has to be noted that P/I null mice are not true ICAM-1 knockouts. The P/I null mice may have had low levels of alternatively spliced forms of ICAM-1 that could have been up-regulated on the vascular endothelium, and thereby promoted neutrophil migration [28,29]. However, this possibility is remote, since the 3E2 mAb that was used in the present study corresponds to the common form of ICAM-1. Further, the lack of ICAM-1, *per se*, is not a critical factor that results in dysfunctional β_2_-integrin-mediated migration. Finally, other adhesion molecule(s), ligand(s), and/or yet unknown counter-receptor(s) might also mediate neutrophil infiltration. For example, ICAM-2, a ligand for β2-integrins, and α4-integrins (α4β1/VLA-4 and α4β1/VCAM-1), could be potential candidates [30-34]. In addition, neutrophils also express CD11d/CD18 and α9-integrin, which both bind to VCAM-1, and could possibly play an important role in neutrophil extravasation, at sites of inflammation [35]. The importance of α4- and α 9-integrin/VCAM-1 pathways in neutrophil infiltration in I/R-induced hepatic injury remains unclear. Further, other proteins are recognized to act as ligands for β_2_-integrins such as those produced during coagulation and complement pathway activation, which could mediate neutrophil adhesion and infiltration into the ischemic liver [36-39]. Therefore, evidence supports this study's finding that ICAM-1 deficiency does not play a key role in neutrophil infiltration and hepatic injury, and that other compensatory mechanisms exist to fulfill the role of ICAM-1.

Inflammatory cytokines such as TNF-α and IL-6 have been shown to play key roles in the pathophysiology of hepatic I/R injury [2,17,40]. TNF-α is the proximal cytokine that is expressed following hepatic I/R, and correlates with hepatic reperfusion injury. IL-6 is a multifunctional cytokine that is both pro-mitogenic and anti-apoptotic for hepatocytes, and is considered a marker for tissue injury severity [41,42]. The data from this study corroborates this as it was found that TNF-α and IL-6 levels paralleled ALT plasma levels (Figure 5A. and 5B). There was no significant difference in plasma TNF-α, and IL-6 levels between the wild-type and P/I null mice.

The CXC-chemokine production was also examined in this study. Plasma MIP-2 and KC levels in the sham groups were constant and minimal, and a significant increase was induced by hepatic I/R in both wild-type and P/I null mice (Figure 5C. and 5D). However, in contrast to the plasma TNF-α and IL-6, a significant difference was observed between the wild-type and P/I null mice CXC-chemokine levels after 6 h of reperfusion. This is a novel observation and the exact mechanism to explain the reduced chemokine production in P/I null mice in response to hepatic I/R is not known, though it may be postulated that the adhesion molecule deficiency may play a role. The genetic knockout mice have altered expression of other molecules which may have reflected the expression of the chemokines. In support of this study, a recent report showed significantly lower chemokine production (i.e. KC) in P/E-selectin deficient mice than their wild-type counterparts [43]. In addition, a recent study highlights the role of selectins and non-integrin collagen receptors in chemokine production and function through p38 mitogen-activated protein kinase and NF-κB activation [44]. Further studies are necessary to examine the role of these adhesion molecules in chemokine regulation and their pathophysiologic role in different organ systems.

Previous studies have suggested a direct association between CXC-chemokines, neutrophil recruitment and liver injury. Specifically, blockage of CXC-chemokines with antibodies was associated with neutrophil infiltration and liver injury in the rat and mouse models of warm hepatic I/R [2,40]. This is in part consistent with the wild-type data presented in this study, in that the CXC-chemokine levels correlated with liver injury and neutrophil infiltration during the early-phase of hepatic I/R (i.e. 6 h of reperfusion). However, during the late-phase of hepatic I/R (i.e. 15 h of reperfusion), the CXC-chemokines were at baseline levels, while neutrophil infiltration was maximal. The neutrophil infiltration may have been mediated by other more potent chemoattractants (e.g. C5a, LTB_4_) and mediators (e.g. apoptotic cells). This hypothesis is supported by Dorman *et al*'s study, in which a CXC-independent neutrophil infiltration into the liver was found in response to apoptotic cells in a mouse model of endotoximia [45]. They showed that wild type as well as the CXCR2 -/- mice had similar neutrophil infiltration and liver injury. There are other potential factors to explain why neutrophil trafficking was not associated with chemokine production. One possible explanation is that the generated CXC-chemokine in P/I null mice was at its optimal concentration to mediate neutrophil infiltration and liver injury. Further, other inflammatory mediators may have been involved in neutrophil infiltration (e.g. C5a, LTB_4_). Finally, the nature of hepatic sinusoidal endothelium, which is fenestrated, may have allowed direct adhesion of neutrophils to the hepatocytes, resulting in liver damage. Future studies are necessary to examine the potential role of these various factors in neutrophil infiltration in hepatic I/R injury.

The survival data presented in this study showed that although not statistically significant the P/I null mice exhibited a favorable trend toward increased survival than their wild-type counterparts. The data also suggested that the potential survival advantage of P/I null mice was not a result of decreased hepatic injury. Since local organ injury appeared to be similar between both groups, it is likely that the P/I null mice were less susceptible to the systemic manifestations of hepatic I/R injury, such as acute respiratory distress syndrome, and multiple organ dysfunction syndrome [46]. It has yet to be elucidated though, whether the decreased CXC-chemokines had a potential role in favoring the survival. A previously published study demonstrated that P-selectin inhibition improved the survival of mice subjected to warm intestinal I/R, in which T lymphocytes (with Th2 profile) played a central role [47]. This is further supported by a study that has implicated CD4+T-lymphocytes as key regulators in I/R-induced inflammatory response in the liver [48]. The profile of Th1 and Th2 cytokines in P/I null mice has not been studied and as such, future studies are warranted to examine the role of T lymphocytes, in their contribution to increased survival.

In summary, the results of this study suggest that P-selectin and ICAM-1 adhesion molecules do not play a critical role in mediating neutrophil infiltration and liver injury caused by hepatic I/R. However, these adhesion molecules may play a role in CXC-chemokine regulation, which may exhibit other functions than chemotactic activities. Inhibition of these adhesion molecules may enhance overall survival by playing a role in the systemic organ injury that often ensues following liver I/R.

## Abbreviations

**ICAM-1**, Intercellular adhesion molecule-1; **I/R**, ischemia/reperfusion; **IL-6**, interleukin-6;**LFA-1**, lymphocyte function-associated antigen; **mAbs**, monoclonal antibodies; **MPO**, Myeloperoxidase; **P/I null mice**, P-selectin/ICAM-1-deficient mice; **TNF**, tumor necrosis factor; **WT**, wild-type.

## Competing interests

The author(s) declare that they have no competing interests.

## Authors' contributions

This study represents parts of the Research Thesis project performed by KM under the direction of EC. KM carried out the surgical operation, collection of samples, analysis and interpretation of the MPO and ALT data, as well as drafting the manuscript. SD participated in the analysis of the cytokine data and the preparation of the manuscript. EC was responsible for conceiving, supervising the design and performance of the project, as well as preparation of the manuscript. All authors read and approved the manuscript.
